# Distinct Functions of the Primate Putamen Direct and Indirect Pathways in Adaptive Outcome-Based Action Selection

**DOI:** 10.3389/fnana.2017.00066

**Published:** 2017-08-03

**Authors:** Yasumasa Ueda, Ko Yamanaka, Atsushi Noritake, Kazuki Enomoto, Naoyuki Matsumoto, Hiroshi Yamada, Kazuyuki Samejima, Hitoshi Inokawa, Yukiko Hori, Kae Nakamura, Minoru Kimura

**Affiliations:** ^1^Department of Physiology, Kyoto Prefectural University of Medicine Kyoto, Japan; ^2^Department of Physiology, Kansai Medical University Hirakata, Japan; ^3^Department of Physiology, Faculty of Health and Sports Science, Juntendo University Chiba, Japan; ^4^Tamagawa University Brain Science Institute Machida, Japan; ^5^Department of Food and Health Sciences, Faculty of Environmental and Symbiotic Sciences, Prefectural University of Kumamoto Kumamoto, Japan; ^6^Division of Biomedical Science, Faculty of Medicine, University of Tsukuba Tsukuba, Japan; ^7^Graduate School of Comprehensive Human Sciences, University of Tsukuba Tsukuba, Japan; ^8^Department of Functional Brain Imaging, National Institute of Radiological Sciences, National Institutes for Quantum and Radiological Science and Technology Chiba, Japan

**Keywords:** monkey, basal ganglia, dopamine, striatum, reward, direct pathway, indirect pathway

## Abstract

Cortico-basal ganglia circuits are critical regulators of reward-based decision making. Reinforcement learning models posit that action reward value is encoded by the firing activity of striatal medium spiny neurons (MSNs) and updated upon changing reinforcement contingencies by dopamine (DA) signaling to these neurons. However, it remains unclear how the anatomically distinct direct and indirect pathways through the basal ganglia are involved in updating action reward value under changing contingencies. MSNs of the direct pathway predominantly express DA D1 receptors and those of the indirect pathway predominantly D2 receptors, so we tested for distinct functions in behavioral adaptation by injecting D1 and D2 receptor antagonists into the putamen of two macaque monkeys performing a free choice task for probabilistic reward. In this task, monkeys turned a handle toward either a left or right target depending on an asymmetrically assigned probability of large reward. Reward probabilities of left and right targets changed after 30–150 trials, so the monkeys were required to learn the higher-value target choice based on action–outcome history. In the control condition, the monkeys showed stable selection of the higher-value target (that more likely to yield large reward) and kept choosing the higher-value target regardless of less frequent small reward outcomes. The monkeys also made flexible changes of selection away from the high-value target when two or three small reward outcomes occurred randomly in succession. DA D1 antagonist injection significantly increased the probability of the monkey switching to the alternate target in response to successive small reward outcomes. Conversely, D2 antagonist injection significantly decreased the switching probability. These results suggest distinct functions of D1 and D2 receptor-mediated signaling processes in action selection based on action–outcome history, with D1 receptor-mediated signaling promoting the stable choice of higher-value targets and D2 receptor-mediated signaling promoting a switch in action away from small reward outcomes. Therefore, direct and indirect pathways appear to have complementary functions in maintaining optimal goal-directed action selection and updating action value, which are dependent on D1 and D2 DA receptor signaling.

## Introduction

Humans and non-human animals adapt behavior based on previous experience, choosing actions followed by rewards and avoiding those followed by unfavorable outcomes. Midbrain dopamine (DA) neurons respond to outcomes that are more or less rewarding than expected by increase or decrease their firing activity (Schultz, 1997, 1998; Reynolds et al., 2001; Fiorillo et al., 2003; Satoh et al., 2003; Nakahara et al., 2004; Bayer and Glimcher, 2005; Enomoto et al., 2011; Eshel et al., 2016). It is widely believed that cortico-basal ganglia circuits play central roles in outcome-based decision making. Striatal projection neurons, called medium spiny neurons (MSNs), appear to encode the reward value of actions by firing rate (Samejima et al., 2005; Lau and Glimcher, 2008). Indeed, firing activities of monkey MSNs prior to action are strongly associated with predicted action reward value based on action–outcome history (Samejima et al., 2005). The flexibility of this activity under changing reward contingencies is thought to depend on DA input, and the striatum is one of the major targets of dopaminergic innervation. Specifically, reinforcement learning models propose that the reward value of actions encoded by MSN activity is modulated based on reward prediction error signals transmitted by DA (Montague et al., 1996; Doya, 2000).

To adapt to a complex environment where action–outcome relationships are probabilistic, trial-by-trial updating of action reward value according to outcome is insufficient and even detrimental for maximizing rewards over an extended period. For example, it is profitable for animals to repeatedly choose a particular action with higher value and ignore rare occasions of undesirable small rewards after the choice (i.e., stay selection of high value action). This choice manner is the best manner when action–outcome relationships are not changed in the environment. On the other hand, action–outcome relationships are actually not so stable and undergo changes in the environment of real world. Therefore, it is also important to detect when the action–outcome relationship changes in the environment and adapt behavior to obtain larger rewards for reward maximization (i.e., flexible changes of action selection). It is likely that stable and flexible action values are encoded on the activity of distinct population of neurons and that stable and flexible selection of actions are differentially guided in the basal ganglia circuit. Indeed, it has been proposed that the direct pathway promotes the intended action, whereas the indirect pathway suppresses unwanted action (Mink, 1996; Hikosaka et al., 2006; Hikida et al., 2010). If a particular action is predicted to be favorable based on action–outcome history, the stable selection of that action regardless of rare negative outcomes would be supported by the direct pathway. On the other hand, if recent action–outcome history predicts a change in reward contingency, a flexible shift in action choice would be supported by the indirect pathway. Direct and indirect pathways are primarily innervated by DA D1 and D2 receptors, respectively (Kombian and Malenka, 1994; Gerfen et al., 1995; Reynolds et al., 2001; Shen et al., 2008). However, it remains unclear if these two pathways and associated DA signaling pathways play different roles in action selection based on reward history.

To assess possible contributions of the direct and indirect pathways to action selection based on action–outcome history, we examined the responses of monkeys during a reward-based probabilistic learning paradigm (Samejima et al., 2005) under control conditions, D1 antagonist local infusion, and D2 antagonist local infusion into putamen. In this task, two alternative choices, lever turn to a left or right target, were associated with predetermined probabilities of large and small reward. When facing a new action–outcome contingency (i.e., larger reward for lever turn in the direction opposite to that on previous large reward trials), the monkeys needed to switch target choice based on trial and error (pre-adaptation stage). Thus, the task requires the integration of action–outcome history over multiple trials within a specific reward schedule. After a certain number of trials in which the action–outcome contingency remains stable, the task requires stable choice of the higher-value target regardless of infrequent small reward outcomes to optimize reward (termed the “post-adaptation” stage). To examine the functions of the two pathways in regulating this balance between flexible and stable action selection, we measured changes in action selection after intra-putamen injection of a D1 antagonist (to disrupt adaptive DA effects in the direct pathway) or a D2 receptor antagonist (to disrupt adaptive DA effects in the indirect pathway). We found that D1 and D2 receptor-mediated signaling mechanisms regulate the balance between stable and flexible action selection to optimize reward.

## Materials and Methods

### Experimental Animals

Experiments were conducted on two Japanese monkeys (*Macaca fuscata*: Monkey A, female, 7.0 kg and Monkey G, female, 7.0 kg). Water intake was controlled while the monkeys took food *ad libitum* during weekdays. On the weekend, they received food and water freely. All surgical and experimental procedures were approved by the Animal Care and Use Committee of Kyoto Prefectural University of Medicine and conducted in accordance with the National Institutes of Health Guidelines for the Care and Use of Laboratory Animals.

Four head-restraining bolts and one stainless steel recording chamber were implanted for recording putamen neuronal activity and local injection of DA D1 and D2 receptor antagonists. The chamber was placed tilting laterally at 45° and aiming at 17 mm anterior, 14 mm lateral and 9 mm above from the interaural line according to Horseley–Clark stereotaxic coordinates (Kusama and Mabuchi, 1970) of the left hemisphere. All surgeries were performed in a sterile operating environment under anesthesia using Ketamine hydrochloride (10 mg/kg, i.m.) and sodium pentobarbital (Nembutal, 27.5 mg/kg i.p.), with supplementary Nembutal (10 mg/kg/h, i.p.) as needed.

### Apparatus

**Figure 1:**
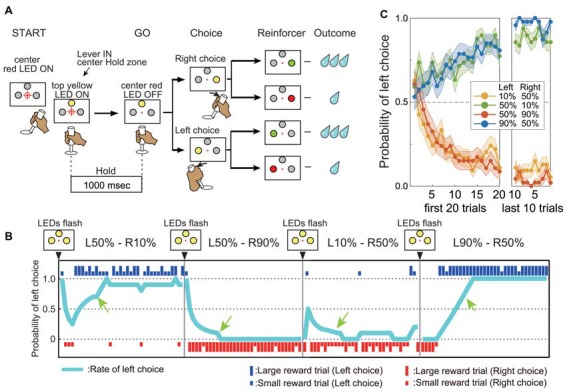
Performance in the reward-based free choice task. **(A)** Time chart of events during the task. **(B)** Representative records of one monkey’s choices and outcomes during four successive action–outcome contingency blocks. Blue and red bars indicate left and right choices, respectively. The long and short bars indicate large and small rewards, respectively. Sky blue line indicates the probability of left choice (see “Materials and Methods” Section). The green arrow indicates the beginning of the “post-adaptation” stage. **(C)** Average mean probability of left choice (±SEM) in four different action–outcome contingency blocks. Data from both monkeys and all blocks before drug injections are shown (54 L10%–R50% blocks, 49 L50%–10% blocks, 46 L50%–R90% blocks and 47 L90%–R50% blocks). The left graph shows the mean probability of left choice for the first 20 trials after changing to a new block. The right graph shows the mean probability of left choice for the last 10 trials of the block.

The monkeys sat on a primate chair facing a steel panel placed 30 cm away. On the panel were embedded three large LEDs (10 mm in diameter, Sunmulon, Tokyo) arranged in a triangle (up, left and right). The distance between left and right LEDs was 11 cm. There was also a small red LED (5 mm in diameter) at the center (Figure 1A). A handle bar for left-right turns was placed 20 cm from the monkey’s body. The left hand of the monkey was physically restrained so that only the right hand was used. The position of the handle was monitored on a laboratory computer (Macintosh G3) through an A-D converter interface at a sampling rate of 100 Hz. Task events and acquisition of behavioral and electrophysiological data were controlled by LabVIEW 5 (National Instruments Co).

### Action Selection Task Based on Action–Outcome History

The monkeys performed a reward-based free choice task (Samejima et al., 2005). The start of a sequence of trial blocks was indicated by the flashing of all LEDs on the panel. Within a block, the start of each trial was cued by illumination of the central small red LED, after which the monkey had to move the handle to the central position within 4.0 s. Proper positioning of the handle was indicated by illumination of the top larger LED in yellow. After holding the handle for 1.0 s (the “Hold” period), the central small red LED dimmed as a GO signal. The monkey was then required to choose either left or right by turning the handle within 5.0 s. After the handle position entered the left or right target zone, a large LED on the chosen side was illuminated in yellow. Further positioning of the handle in the target zone for 0.5 s led to a change in LED color from yellow to either green or red according to pre-programmed probabilities. The green light indicated a large reward (0.2 mL water) and red indicated a smaller reward (0.07 mL). To study flexible updating of choice behavior based on target value, we manipulated the probability of a large reward associated with each target in different trial blocks. In the “L90%–R50%” block for example, the probability of a large reward was 90% for the left turn and 50% for the right turn. We used four asymmetrically rewarded trial blocks: “L90%–R50%”, “L50%–R90%”, “L50%–R10%” and “L10%–R50%”. The target-reward probability contingency was fixed for a block of 30–150 trials and then changed. All large LEDs flashed three times before starting a new block. When a monkey chose the target with higher large reward probability (higher-value target) on not less than 7 of the previous 10 trials, we considered that the monkey had “adapted” its choice to the current contingency (post-adaptation stage: after green arrows in Figure 1B). If the probability of choosing the higher-value target (ratio of higher-value choices to trials) remained above 70% for 20 trails (Monkey A) or 25 trails (Monkey G), the action–outcome contingency was changed. Thus, the monkeys had to adjust their choices to maximize reward gain by trial and error (Figures 1B,C) after the change of block. Failures of acquisition and of holding the central bar at the target zone were regarded as errors and excluded from further analysis. Monkey A had performed this task for 36 months and Monkey G for 12 months before these studies began.

### Dopamine Receptor Antagonist Injection

**Figure 2:**
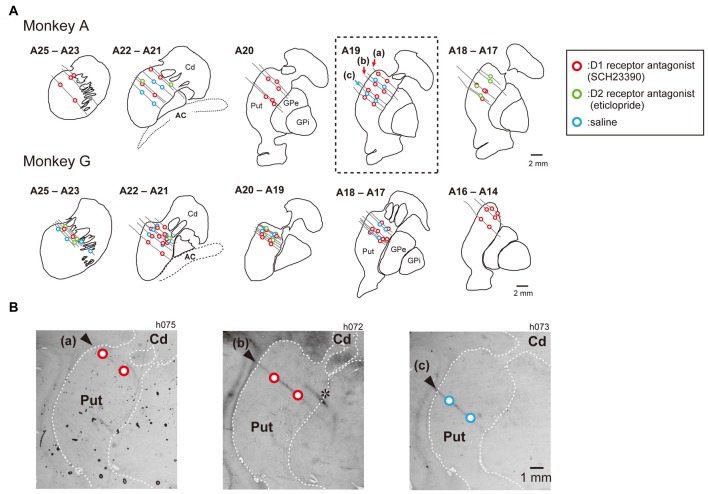
Injection sites of SCH23390, eticlopride and saline in the putamen. Histologically reconstructed frontal sections of the left hemisphere putamen (Put) and caudate nucleus (Cd) from rostral to caudal level of the Horsley–Clark atlas. **(A)** Circles are injection sites of SCH23390 (red), eticlopride (green) and saline (blue). Gray lines indicate track of injection tube observed in nissl-stained section. **(B)** Nissl-stained frontal section of at the level of A19 (box of dotted line in **A**) of the Horsley–Clark atlas (Monkey A). *Asterisk* indicates electrolytic lesion mark made by passing DC current (30 μA for 20 s) through a tungsten microelectrode.

Before drug-injection experiments, we performed single unit recordings to map out the caudate and putamen. A total of 55 recording tracks were performed in Monkey A and 43 in Monkey G. The putamen and caudate were identified by characteristic discharges of phasically active neurons and tonically active neurons. The recorded and injected sites were later verified histologically (Figure 2). The number of examined block type is summarized in Table 1. For drug injections, we used a stainless steel tube (o.d., 0.3 mm; i.d., 0.17 mm) connected by a Teflon tube (o.d., 0.92 mm; i.d., 0.46 mm) to a Hamilton syringe (5.0 μL). A Teflon-coated tungsten wire electrode (A-M Systems, Inc.) was threaded into the injection tube to confirm tip location by single- or multi-unit recordings. The injection tube was lowered by a micromanipulator (MO-95, Narishige, Tokyo, Japan) through a Stainless steel guide tube (o.d., 500 μm) penetrating the cortex over the putamen to a depth of around 5 mm below the dura mater. At each injection site, drug solution (0.8 μL) was pressure-injected by an injection pump (BAS Inc., MD-1001) at 0.1 μL/min. We used SCH23390 (8 μg/μL in saline) as the DA D1 receptor antagonist and eticlopride (6 μg/μL in saline) as the DA D2 receptor antagonist (Reynolds et al., 2001; Bari and Pierce, 2005; Sigma, St. Louis, MO, USA). These doses were chosen based on previous reports (Watanabe and Kimura, 1998; Nakamura and Hikosaka, 2006). We also injected saline vehicle in a separate experiment to ensure that any changes in behavior were not caused by the mechanical influences of liquid injection on brain tissue. We usually injected drug solution or saline twice, at 2 mm above the ventral border and 2 mm under the dorsal border of the putamen. If the putamen was thinner than 4 mm across a particular injection path, however, we injected the drug only once at the center. Task performance was measured over the 30 min following injection.

**Table 1:** Number of trial blocks examined for behavioral effects of SCH23390, eticlopride and saline injections.

	Option pairs Left Right	SCH23390	Eticlopride	Saline
Animal A	90%–50%	6	3	1
	50%–10%	1	3	1
	50%–90%	4	4	2
	10%–50%	1	3	2
Animal G	90%–50%	24	12	21
	50%–10%	29	10	12
	50%–90%	25	11	14
	10%–50%	34	11	18

*% denotes probability of reward*.

### Data Analysis

**Figure 3:**
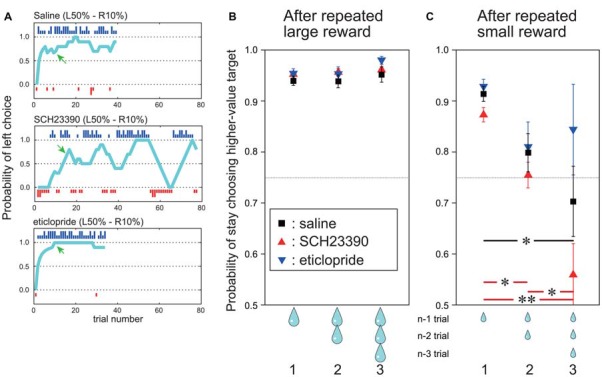
During the post-adaptation stage, SCH23390 and eticlopride had distinct effects on reward-oriented choices. **(A)** Probability of left choice during a single block following saline injection (top), SCH23390 injection (middle) and eticlopride injection (bottom). Other symbols are the same as Figure 1B. **(B)** Mean probability of stay choosing the higher-value target (*y* axis) after one, two and three successive trials with large reward outcome under each injection condition. Red triangles indicate SCH23390, blue triangles eticlopride and black squares physiological saline injection. Data are based on all trials choosing the higher-value target after reaching the post-adaptation stage level (70% level in last 10 trials). n: current trial number. **(C)** Mean probability of stay choosing the higher-value target after successive small reward outcomes for each injection condition. Vertical bars indicate SEM. Red and black horizontal bars indicate significant differences among number of successive small rewards for SCH23390 and saline injection conditions. ^*^p < 0.05, ^**^p < 0.001, *post hoc* test (Tukey–Kramer tests).

In the present study, we focused our analyses on performance in the post-adaptation stage (see “Action Selection Task Based on action–outcome History” Section). We defined the period before reaching the post-adaptation stage as the “adaptation stage”. To assess choice stability for the higher-value target, we calculated the proportion of times (probability) the animal chose the higher-value target after a given number of consecutive large (Figure 3B) or small (Figure 3C) rewards, referred to as the probability of stay choosing higher-value target.

The probability of stay choosing higher-value target was analyzed by two-way analysis of variance (ANOVA) with factors drug type (SCH23390, eticlopride, or saline) and number of consecutive large or small rewards (1, 2 or 3) as outcome. Tukey–Kramer tests were used for *post hoc* comparisons (Figures 3B,C).

## Results

In a free choice task requiring monkeys to turn a handle left or right to obtain a higher-value reward (larger water volume) with asymmetrically assigned probability within trial blocks (e.g., higher probability of the larger reward for a left turn), both monkeys were far more likely to choose the response associated with the high-value reward. Furthermore, both monkeys quickly adapted their choices to a new action–outcome contingency in the next block of trials. In the example shown in Figure 1B, the monkey quickly learned to choose the left target in L50%–R10% blocks (i.e., 50% chance of large reward for left turn, 10% probability of large reward for right turn). In the next block, the large reward probability for the left target remained 50% while the large reward probability for the right target was changed to 90% (L50%–R90% condition), and the monkey quickly switched to the right target on most trials (Figure 1B). Both animals learned to choose the higher-value target depending on the *relative* difference in large reward probability (Figure 1C). Further, once the monkeys learned to choose the higher-value target, they kept making consistent choices regardless of occasional small rewards. For example, although the monkey could receive a large reward for a right turn and a small reward for a left turn in L50%–R10% blocks (in 10% and 50% of trials, respectively), they kept choosing the left target with higher large reward probability in the post-adaptation stage (Figure 1B, probability of left choice after green arrows “post-adaptation stage” in L50%–R10% and L10%–R50% blocks). Thus, the monkey’s choice was based on the long-term action–outcome history rather than the outcome of recently chosen action. Similar response patterns were observed after physiological saline injections in the putamen. As shown in the example (Figure 3A, top), once the “adapted” to choose the target associated with higher large reward probability was established (defined as large reward target choice in 7 out of the preceding 10 trials, see “Materials and Methods” Section), the monkey kept choosing the same target until the reward schedule changed in the next block.

To analyze the effect of outcome on choice behavior in this post-adaptation stage, we computed the probability of stay choosing the higher-value target after receiving a large reward once, twice and three times consecutively (termed the “stay” choice; Figure 3B). After saline injections, the mean probability of stay choosing the higher-value target after receiving a large reward was already 95%, and it stayed high after receiving two or three consecutive large rewards (Figure 3B, black squares). On the other hand, the probability of stay choosing the higher-value target after a small reward on the preceding trial was slightly reduced to 91%, and decreased further to 80% if the two preceding trials yielded small rewards and to 70% if the three previous trials yielded small rewards. This indicates that the monkeys tended to switch their choice after receiving multiple consecutive small rewards (Figure 3C, black squares).

Infusion of DA receptor antagonists markedly altered these choice patterns. After injection of the D1 antagonist SCH 23390, the choice of the higher-value target reached the post-adaptation stage within a block once. However, the following choice behavior fluctuated (Figure 3A, middle row). On the other hand, after injection of the D2 antagonist eticlopride, which is expected to disrupt DA-dependent adaptive changes in the indirect pathway, the probability of choosing the higher-value target remained high, and appeared even higher and more stable than after saline injections (Figure 3A, bottom row).

To characterize these behavioral changes quantitatively, the probability of stay choosing the higher-value target after receiving a large reward or a small reward one, two and three times in succession were compared among saline, SCH 23390, and eticlopride injection conditions (Figures 3B,C). The probability of stay choosing the higher-value target after receiving a large reward remained high for both antagonists and did not differ from the saline condition. Two-way ANOVA indicated that the probability did not differ significantly by drug type (*F*_(2,720)_ = 1.99, *p* = 0.14), number of preceding successive large reward trials (*F*_(2,720)_ = 2.20, *p* = 0.11), or the interaction between drug type and number of preceding successive large rewards (*F*_(4,720)_ = 0.29, *p* = 0.88; Figure 3B).

By contrast, the probability of stay choosing the higher-value target after consecutive small rewards in the post-adaptation stage changed significantly following antagonist infusion, and the changes were distinct for SCH23390 and eticlopride. Following SCH23390 injection, the mean probability of stay choosing the higher-value target was only 56% after three consecutive small rewards (vs. 70% for saline; Figure 3C, red triangles). Following eticlopride injections, however, the probability of stay choosing the higher-value target was 84% after three consecutive small rewards (higher than in the saline condition; Figure 3C, blue triangles). ANOVA revealed that the probability of stay choosing the higher-value target after small reward was significantly altered by the DA receptor antagonist type (*F*_(2,461)_ = 26.6, *p* < 0.001), the number of preceding successive small reward outcomes (*F*_(2,461)_ = 10.81, *p* < 0.001), and by the interaction of antagonist type and the number of preceding successive small reward outcomes (*F*_(2,461)_ = 2.75, *p* < 0.05). *Post hoc* Tukey–Kramer tests showed that as the number of preceding successive small rewards increased, the probability of stay choosing higher-value target was decreased (*p* < 0.05) significantly by SCH23390 injection. This effect was also observed after saline injections (Tukey–Kramer test, *p* < 0.05). After eticlopride injection, however, the probability of stay choosing the higher-value target remained high (84%) even after receiving three consecutive small rewards. Following eticlopride injection, *post hoc* Tukey–Kramer tests revealed that the probability of stay choosing higher-value target after one, two, and three successive small rewards was not significantly influenced. We also conducted this same analysis during the pre-adaptation stage, but found no significant effect of antagonist type or number of preceding successive large and small rewards.

## Discussion

These differential effects of DA D1 and D2 receptor antagonists on performance of a free choice task with probabilistic reward strongly suggest that direct pathway D1 receptor signaling promotes behavioral stability to attain high-value rewards while indirect pathway D2 receptor signaling facilitates switching away from smaller reward outcomes. Although D1 and D2 receptor antagonists had reciprocal effects, D1 antagonism leading to choice instability and D2 antagonism to enhanced stability despite successive small reward outcomes, both changes resulted in suboptimal reward-based action selection. Therefore, D1 and D2 signaling mechanisms in direct and indirect basal ganglia pathways appear to act cooperatively to optimize reward by regulating the balance between stable and flexible responses under conditions of probabilistic reward contingencies.

Antagonism of D1 receptors facilitated switching from higher-value action to lower-value action after small reward outcome, while the D2 antagonist suppressed switching despite successive small reward outcomes. The response to D1 antagonist infusion strongly suggests that D1 receptor-mediated signaling processes are necessary for stable action reward value coding. Modulation of DA signaling in rodents, primates and humans alters behavioral and neural processes based on the action–outcome contingencies (Nakamura and Hikosaka, 2006; Dodds et al., 2008; Shen et al., 2008; Boulougouris et al., 2009; Clatworthy et al., 2009; Cools et al., 2009; Rutledge et al., 2009). The DA D1 receptor-mediated signaling is required for the induction of long-term potentiation (LTP) of cortico-striatal synapses on MSNs (Reynolds et al., 2001; Calabresi et al., 2007). Therefore, D1 antagonism should suppress synaptic strengthening associated with rewarded action.

DA D2 receptor antagonist injection evoked the opposite response to D1 receptor antagonist injection. The “stay” probability after repeated small rewards remained high or even higher than after saline injection during the post-adaption stage. This implies D2 receptor signaling may be crucial for choice switching to avoid unfavorable (small reward) outcomes. Unexpected absence of reward reduces firing rate of DA neurons, which acts as a negative prediction error signal (Schultz, 1998; Satoh et al., 2003; Bayer and Glimcher, 2005). The indirect pathway suppress unwanted action (Mink, 1996; Hikosaka et al., 2006; Kreitzer and Malenka, 2007) and D2 receptor activation is involved in the induction of long-term depression at cortico-striatal synapses (Surmeier et al., 2007; Shen et al., 2008). Thus, DA D2 receptor-mediated signaling appears to promote changes in behavior based on small rewards rather than updating value based on large reward outcomes.

### Dopamine D1 and D2 Receptor-Mediated Adaptive Action Selection in the Striatum

It has been proposed that DA D1 and D2 receptor-mediated signaling mechanisms are differentially involved in encoding action–outcome history (Frank et al., 2004; Lee et al., 2015). When an action outcome is better than expected, the cortico-basal ganglia pathway is thought to be strengthened through D1 receptor-mediated processes such as LTP (Houk et al., 1995; Doya, 2000), which facilitates choice stability and attainment of larger rewards in the long-term. Our results also revealed that D2 receptor-mediated processes maximize rewards by suppressing choices that lead to unfavorable outcomes. After D1 antagonist injection, the probability of stay choosing the higher-value target decreased even in the post-adaptation stage when the monkey received a small reward two and three times in succession. This suggests that the value of the target with higher value was not fully updated during the pre-adaptation stage when D1 receptors are blocked. A similar effect was also observed after saline injection when the monkey received a small reward three times in succession, although the probability of stay choosing higher-value target was still greater than in the D1 antagonist injection condition. In contrast, no such decrease in higher-value target choice was observed after D2 antagonist injection, suggesting that D2 receptor-mediated signaling processes are still operative after reaching the post-adaptation stage in the control condition. Collectively, these findings suggest that D1 signals of higher-value targets estimated from a prolonged action–outcome history may be encoded by D1 receptor processes.

We conclude that DA D1 receptors encode higher-value targets after a prolonged action–outcome history. On the other hand, action switching following unexpectedly small reward outcomes may be modulated by a D2 receptor-mediated process. Thus, complementary roles of direct and indirect pathways may be essential to achieve optimal balance of behavioral stability and flexibility for adaptive action selection.

## Author Contributions

YU designed the study, conducted experiments, analyzed data and wrote the manuscript. KY conducted experiment and analyzed data. KS and AN wrote computer programs for experiments and analyzed data. NM, KE, HY, HI, YH and KN discussed data and manuscript. MK designed the work and discussed data and manuscript.

## Conflict of Interest Statement

The authors declare that the research was conducted in the absence of any commercial or financial relationships that could be construed as a potential conflict of interest.
